# Role of AI and digital pathology for colorectal immuno-oncology

**DOI:** 10.1038/s41416-022-01986-1

**Published:** 2022-10-01

**Authors:** Mohsin Bilal, Mohammed Nimir, David Snead, Graham S. Taylor, Nasir Rajpoot

**Affiliations:** 1 Tissue Image Analytics Centre, Department of Computer Science, University of Warwick, Coventry, UK; 2 Department of Pathology, University Hospitals Coventry and Warwickshire NHS Trust, Coventry, UK; 3 Warwick Medical School, University of Warwick, Coventry, UK; 4 Institute of Immunology and Immunotherapy, University of Birmingham, Birmingham, UK; 5 The Alan Turing Institute, London, UK

**Keywords:** Colorectal cancer, Predictive markers

## Abstract

Immunotherapy deals with therapeutic interventions to arrest the progression of tumours using the immune system. These include checkpoint inhibitors, T-cell manipulation, cytokines, oncolytic viruses and tumour vaccines. In this paper, we present a survey of the latest developments on immunotherapy in colorectal cancer (CRC) and the role of artificial intelligence (AI) in this context. Among these, microsatellite instability (MSI) is perhaps the most popular IO biomarker globally. We first discuss the MSI status of tumours, its implications for patient management, and its relationship to immune response. In recent years, several aspiring studies have used AI to predict the MSI status of patients from digital whole-slide images (WSIs) of routine diagnostic slides. We present a survey of AI literature on the prediction of MSI and tumour mutation burden from digitised WSIs of haematoxylin and eosin-stained diagnostic slides. We discuss AI approaches in detail and elaborate their contributions, limitations and key takeaways to drive future research. We further expand this survey to other IO-related biomarkers like immune cell infiltrates and alternate data modalities like immunohistochemistry and gene expression. Finally, we underline possible future directions in immunotherapy for CRC and promise of AI to accelerate this exploration for patient benefits.

## Introduction

Colorectal cancer (CRC) is the second most common cause of cancer-related death in the UK [1]. Some of these deaths may be avoided if cancer progression or recurrence can be predicted early and treated. Personalised medicine makes use of individual-specific information—such as their genetic information—to tailor the diagnosis and treatment of cancer. In CRC, however, personalised treatment options are limited to only a subset of patients. For example, certain molecular biomarkers, such as the mismatch repair (MMR) status and NRAS mutations, have a major influence on how CRC patients can be managed [2]. The remarkable promise of immunotherapy in metastatic MMR deficient (dMMR) or microsatellite instability-high (MSI-H) cancer is highlighted by a subset of CRC patients who achieve long-term durable remissions [3], with MSI-H tumours possessing more neoantigens thus being more visible to the immune system and more responsive to immune-checkpoint inhibitor therapy [4–6].

Our main focus in this paper is on the histopathology-based literature on immunotherapy in CRC. In the next section, we introduce MMR proteins and the impact of their inactivation? We have further discussed two fundamental questions concerning the immune system and tumour microenvironment (TME): (1) how does the immune system respond to tumours? (2) How can the immune system be harnessed to fight tumours? We discuss the significance of the immunological phenotype of microsatellite instability (MSI) in deciding on immunotherapy treatment for CRC tumours in the “Immunotherapy in CRC” section. To conclude this part of the literature, we briefly describe the alternative immunotherapeutic approaches, cytokines, oncolytic viruses, tumour vaccines, and other cells in the TME which are found relevant to immunotherapy in CRC.

The AI for immunotherapy survey is also focused on studies which include histopathology data for therapeutic decision-making. It provides the generic and fundamental concepts of artificial intelligence (AI) and how it gets integrated into histopathology. Recently, AI and machine learning have appeared as novel tools for predicting and evaluating actionable genetic alterations from routine histology images. It has also been a gateway to a new research approach, particularly AI for immunotherapy from within the horizon of computational pathology. This led to modelling the prediction of MSI-H tumours and other molecular pathways, subtypes, and genetic mutations of several cancer types, including CRC. In the final section, we discussed the key takeaways from this rapid progress of AI for immunotherapy in CRC and potential future directions.

## Immune system and TME

This section describes four key concepts related to immunotherapy in CRC.

### What are mismatch repair (MMR) proteins?

Cancer arises by mutations in the cellular genome. Colorectal tumours from different patients vary markedly in their mutational burden [7]. An important cause of mutations in colorectal cancer, accounting for about 15% of cases of non-metastatic CRC, is deficiency or inactivation of the DNA mismatch repair (MMR) pathway [8]. This pathway is essential for genome stability, correcting mismatched DNA nucleotides arising from polymerase errors during DNA replication or from chemical damage [9]. The most important genes involved in MMR are MLH1 (human mutL homolog 1), MSH2 (human mutS homologue 2) MSH6 (human mutS homologue 6) and PMS2 (human postmeitotic segreagation 2). Inactivation of these genes can occur sporadically, for example, through hypermethylation of the MLH1 gene [10], or can be inherited (e.g., Lynch syndrome).

### What happens when MMR proteins are inactivated?

Inactivation of the MMR pathway, whether inherited or sporadic, leads to a high frequency of mutations throughout the genome. Microsatellites are small repetitive stretches of DNA sequence scattered throughout the genome that are prone to mutation. Loss of MMR function therefore causes high-level microsatellite instability (MSI-H) creating differently sized repeat sequences, not found in the normal DNA. These repeat sequences can be detected by polymerase chain reaction assays or genome sequencing; their presence therefore indicates MMR dysfunction with concomitant high mutational burden spread across the whole genome [11, 12]. Alternatively, loss of the key MMR genes can be detected by immunohistochemistry (IHC).

### How does the immune system respond to tumours?

The immune system employs multiple mechanisms to differentiate between self (e.g., normal tissues) and non-self (e.g., virus-infected cells) to avoid attacking the former. Although cancer cells arise from normal cells, they express proteins that, in some cases, are sufficiently altered to be recognised by the immune system as non-self and thus targeted by the immune system. Some of these proteins are aberrantly expressed proteins not usually made by adult cells (such as carcinoembryonic antigen, a protein normally expressed only during foetal development). Others are created de novo by mutations within the cancer cell genome altering the amino acid sequence of proteins. These neoantigens are highly attractive targets for immunotherapy. First, their absence from normal tissues reduces the risk of off-target effects from immunotherapies seeking to target them. Second, they are highly foreign to the immune system allowing them to be efficiently recognised [13]. Potential limitations of using neoantigens for cancer immunotherapy are first, the fact that most are unique to an individual patient and secondly because their generation is stochastic: only a small proportion of genomic mutations lead to the formation of a neoantigen able to be displayed by the cancer cell and then recognised by the immune system. Tumours with a high mutational burden, such as MSI-H CRC, are therefore simply more likely to contain neoantigens than lower mutational burden tumours such as microsatellite stable (MSS) CRC.

The fact that tumours expressing immunogenic tumour antigens or neoantigens can persist and grow in patients with overtly normal immune function indicates other processes must be operating to prevent tumour eradication. Tumours comprise of malignant cells but also a diverse range of non-malignant stromal and immune cells that collectively form the tumour microenvironment (TME). Some of these immune cells are potentially capable of initiating or exerting anti-tumour activity: these include dendritic cells, CD8+ T cells, Th1 CD4+ T cells, natural killer cells, M1 macrophages and N1 neutrophils [14]. Accordingly, increased intra-tumoural numbers of some of these cells are associated with improved prognosis for several cancers including colorectal cancer [15]. Conversely, other immune cell types, such as regulatory CD4+ T cells and myeloid-derived suppressor cells support the growth and maintenance of the tumour, limiting anti-tumour immunity within the TME. These cells may also support tumour growth by secreting factors that promote angiogenesis, the formation of new blood vessels essential for tumours to grow beyond the limits of oxygen diffusion, and promote tumour cell metastasis.

It is important to note that this balance between anti- and pro-tumour immunity is not static and changes over time, a process called cancer immunoediting[16]. During the first phase, elimination, transformed cells are recognised by the immune system and eliminated. If successful, then the individual will not develop a clinically apparent tumour. However, if elimination is unsuccessful, then the second phase, equilibrium, commences with the anti-tumour cells limiting the growth of the transformed cells but being unable to eliminate them. Based on the long period between carcinogen exposure and cancer development [17] the equilibrium phase likely persists for decades [16]. Further evidence for long-term equilibrium comes from unfortunate cases of cancer transmission by organ transplantation. Here, transplant recipients developed cancers after receiving organs harvested from donors who had previously recovered from cancer, in some cases over a decade before transplantation occurred [18]. Equilibrium may end with the immune system clearing the transformed cells. However, it is a dynamic process, and alternatively, the transformed cells may evolve to evade immune-mediated equilibrium to enter the third phase and escape, producing a tumour.

### How can the immune system be harnessed to fight tumours?

Cancer that develops in immunocompetent animals, having evolved under immune pressure, are less well recognised by immune effectors [19]. Cancer cells from patients similarly show the effects of immune escape, for example, downregulating expression of human leucocyte antigen (HLA) class I molecules and other components of this pathway essential for recognition by CD8+ T cells [20]. Nevertheless, compelling results from multiple Phase III clinical trials and the licensing by regulatory authorities of several immunotherapies able to produce durable clinical responses show it is possible to restore immune control of cancer for some diseases and patients [21].

**Fig. 1:**
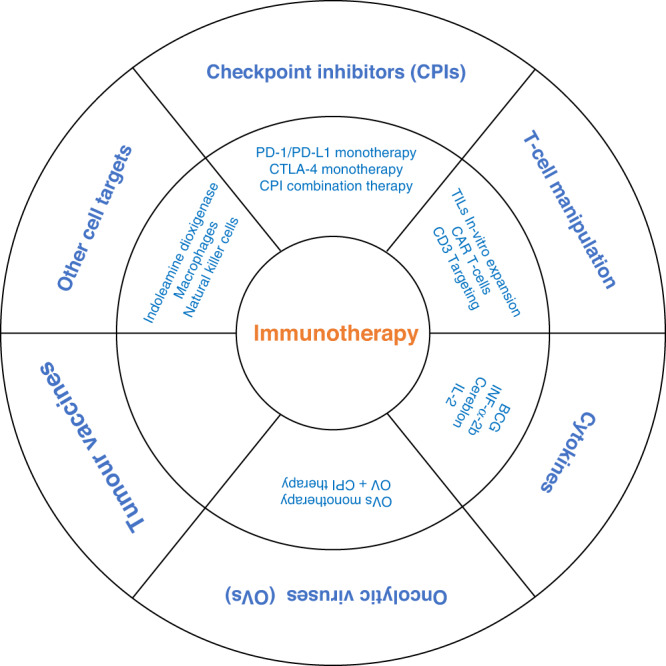
A summary of major immunotherapeutic approaches. This include Checkpoint inhibitors (CPIs), T-cell manipulation, cytokines, oncolytic viruses (OVs) and tumour vaccines.

Several avenues are being taken to explore how to control tumours using the immune system. These include checkpoint inhibitors, T-cell manipulation, cytokines, oncolytic viruses and tumour vaccines. A summary of these is provided in (Fig. 1). In the next section, we describe immunotherapy approaches in CRC followed by AI for CRC immunotherapy, and a discussion to synthesise the reviewed literature, limitations and advantages, and future potential of AI for immunotherapy in CRC.

## Immunotherapy in CRC

As of 2022, two types of immunotherapy agents have been approved by the United States Food and Drug Administration (FDA) for use in CRC patients with MSI-H tumours [4]. A useful paradigm for understanding how these immunotherapies work is the cancer immunity cycle [22]. In this model, tumour antigens from tumour cells are internalised by antigen-presenting cells and displayed to T cells in draining lymph nodes. This stimulates tumour antigen-specific T cells which then traffic to the tumour and kill tumour cells. Tumour cell death then releases tumour antigens that can prime new tumour-specific T-cell responses.

The first type of immunotherapy agent licensed for CRC are three different antibodies (Pembrolizumab from Merck, Dorstalimab from Glaxo Smith Klein and Nivolumab from Bristol Meyers Squibb) that act by blocking the programmed cell death protein 1 (PD1) immune checkpoint. The normal role of this checkpoint is to protect host tissues by limiting immune-mediated damage at sites of inflammation. The binding of PD1 on the surface of T cells to its ligand PD-L1 affects T-cell receptor signalling differentially affects different T-cell types. PD1 engagement on effector T cells promotes their apoptosis, anergy and exhaustion, whereas PD1 engagement on regulatory T cells stimulates their proliferation [23]. Consequently, this checkpoint is frequently harnessed by tumours to evade immune-mediated destruction [24]. Antibodies that bind to PD1 or PD-L1, preventing their interaction, therefore act at the final effector stage of the cancer immunity cycle, restoring effector T-cell function within the tumour. For some cancer patients this alone is sufficient to restore immune-mediated control of the tumour, with compelling evidence of efficacy from multiple clinical trials in MSI-H CRC patients [4].

The second type of immunotherapy agent licensed for CRC is an antibody (Ipilimumab, from Bristol Meyers Squibb) that targets a different immune checkpoint called Cyotoxic T-lymphocyte antigen 4 (CTLA4). Expressed on the surface of activated T cells, CTLA4 competes for binding to B7 molecules on the surface of antigen-presenting cells, inhibiting the early priming stage of the T-cell response. Because CTLA4 acts is a different stage of the cancer immunity cycle compared to PD1, inhibiting both checkpoints is synergistic, with anti-PD1/anti-CTLA4 combination therapy yielding improved clinical benefit relative to anti-PD1 monotherapy [5].

### MSI-H CRC tumours have a distinct immunological phenotype

MSI-H tumours are distinct from similarly staged MSS tumours in several respects [25]. They are predominantly located in the right side of the colon, are less likely to metastasise and are less sensitive to 5-fluorocil-based chemotherapy used for first-line therapy of CRC [26, 27]. Their TME is also different. MSI-H CRC tumours are highly infiltrated with T cells with an activated cytotoxic phenotype [28–30]. These effectors are counterbalanced by a range of highly upregulated immune checkpoints that include PD1/PD-L1 and CTLA4 but also Lymphocyte Activation Gene 3 (LAG3), and indolamine 2’3’-dioxygenase (IDO), which inhibits T-cell function by depleting tryptophan levels within the TME [31]. This combination of increased immune effector infiltration and immune inhibitory pathways is consistent with highly mutated MSI-H tumours possessing more neoantigens (thus being more visible to the immune system) and more responsive to immune-checkpoint inhibitor therapy [4–6].

### Alternative immunotherapeutic approaches for CRC

Advances in genetic engineering now make it possible to express conventional or modified T-cell receptors in T cells, redirecting them to recognise new targets. Chimeric antigen receptor T cells (CAR-T cells) are patient-derived T cells that are genetically modified outside the body to express a fusion protein (hence the term chimeric) comprising an intracellular signalling domain and the antigen-binding region of an antibody specific for tumour-associated antigens expressed on the outer surface of the cancer cell [32]. A major advantage of this strategy is that it is not dependent on the cancer cell expressing HLA molecules nor a functional antigen processing pathway; both are frequently downregulated in cancer cells to escape surveillance by conventional T cells. Amongst the targets being explored for CRC therapy is CEA, which as described earlier is absent from normal adult cells but frequently expressed by CRC cancer cells. A phase dose escalation trial of CEA-targeting CAR-T cells in patients with metastatic CEA-positive CRC reported tumour shrinkage in two of the ten patients who received the CAR-T cells with another seven patients achieving stable disease that for two patients were sustained for over 30 weeks [33].

Genetic manipulation of T cells is a complex, expensive process. An alternative, more scalable approach is to use bispecific T-cell engager (BiTE) antibodies: These antibodies are made by fusing two different single chain antibodies such that the new antibody possesses a variable region that recognises the tumour antigen and another able to bind to the T-cell marker CD3 and trigger T-cell activation [34]. A potential disadvantage of the approach is the lack of T-cell specificity compared to some of the other immunotherapy methods, since CD3 is expressed not only by cyotoxic T cells but also by regulatory T cells. Cibisatamab (CEA-TCB) is a BiTE antibody specific for CEA [35] currently being tested for safety and efficacy in a Phase 1b clinical trial (ClinicalTrials.gov identifier: NCT03866239). A potential limitation of this and the CAR T-cell response described above is the use of CEA as a target. Because CEA is not essential for tumour cell survival, resistance can develop due to selection for tumour cells with low or absent expression of CEA. In a recent in vitro study, patient-derived CRC organoids developed resistance to Cibisatamab [36]. Combination approaches may therefore be important to avoid treatment resistance developing when non-essential tumour antigens are being therapeutically targeted.

### Cytokines, oncolytic viruses, tumour vaccines and targeting other cells in the TME

The immune system uses a diverse range of cytokines to co-ordinate immune responses. Non-specific immune stimulation with cytokines has been used to treat patients for decades, although response rates for cytokine monotherapy are modest and toxicity is often dose-limiting [37]. Cytokines are likely more valuable when used in combination with other immunotherapy modalities [38]. Examples include interleukin-2 (IL-2, which supports the growth and differentiation of T cells) and granulocyte-macrophage colony-stimulating factor (GM-CSF, which facilitates processing and presentation of antigens by antigen-presenting cells), both of which have been used in combination with multiple agents.

Oncolytic viruses (OVs) can act as anti-tumour agents in a myriad of ways [39]. They can function via direct oncolysis of the tumour cells but also through other mechanisms that include: serving as vehicles for cytokine expression within the TME, providing activation signals that promote a more hostile TME, or promoting MHC-I-associated presentation of tumour-specific antigens. OVs can also be modified to preferentially infect cancer cells. Given the frequent upregulation of immune checkpoints within tumours, a rational approach is to administer OVs in combination with CPIs, with the former promoting antigen release to stimulate immune effectors and the latter allowing those effectors to function effectively. Since both agents are acting at complementary stages of the cancer immunity cycle, this strategy is likely to be synergistic [40, 41].

An alternative approach to stimulate anti-tumour effectors, which like OVs could be used in combination with checkpoint inhibitors to achieve synergy, is therapeutic cancer vaccination. Several clinical trials are currently underway seeking to develop therapeutic vaccines to treat CRC and/or prevent recurrence. A diverse range of modalities are being investigated, including dendritic cells loaded with CEA, EpCAM, p53 or 5T4 antigens (all of which are potential immune targets in CRC) or tumour cell lysates [42]. Like OVs, these vaccines can potentially be combined with immune-checkpoint inhibitors as a rational combination strategy.

In the next section, we describe the fundamental of Artificial Intelligence (AI) and then we will present our survey of literature on AI for immunotherapy in CRC.

## Artificial intelligence and immunotherapy in CRC

### Artificial intelligence overview

**Fig. 2:**
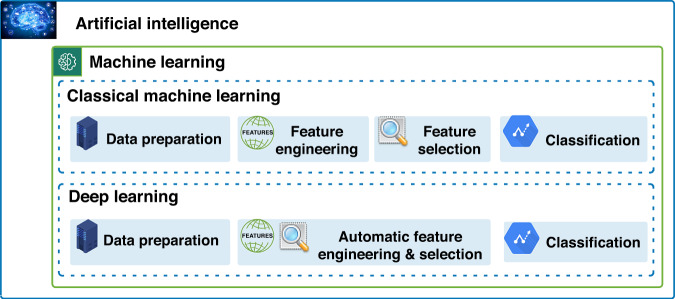
Artificial intelligence and machine learning. Computational workflow for conventional machine learning and deep learning-based modelling.

In general, artificial intelligence (AI) refers to machines designed to imitate human intelligence while performing complex tasks without human intervention. Machine learning (ML) is a branch of AI and computer science that provides various algorithms of learning from the data and examples without being programmed explicitly. Logistic regression, decision trees, random forests, support vector machines, and artificial neural networks (ANN) are few widely used and popular algorithms of classical ML. Deep learning (DL) is a relatively new offshoot of machine learning, where ANNs are designed with several intermediate (or hidden) layers of artificial neurons between the input and output layers and new set of operations as artificial neurons e.g., convolution in convolutional neural networks (CNNs). In this survey, our focus is on both classical ML and DL, which are widely used in computational pathology for a range of related applications in cancer diagnosis and prognosis. Broadly, machine learning refers to models that learn from the data or examples either with or without supervision. Figure 2 illustrates the difference of modelling ML and DL workflows for AI systems.

A general workflow of modelling artificial intelligence by machine learning involves finding the discriminative patterns within the data that involves data preparation, feature engineering, feature selection and classification or regression (classification refers to prediction of discrete labels, whereas regression refers to continuous labels), where learning is managed by training and validating/testing the problem-solving model. Classical ML refers to the manual or hand-crafted feature engineering and selection of most relevant features, whereas the modern deep learning (or DL) methods employ data-driven feature engineering and selection intrinsically. We will refer the classical ML as ML from hereon. In ML, the term feature engineering refers to encoding the raw data in meaningful measures and descriptors using domain knowledge and expertise for better representation of data to facilitate learning. Feature selection is the process of selecting subset(s) of relevant features by removing irrelevant and redundant features. The blend of ML and DL is gaining popularity in digital and computational pathology, where DL is mainly used for learning representation of pixel data in histology images and ML for learning decision boundaries or classifications. Conventionally, ML was often divided into three subcategories: supervised, unsupervised and reinforcement learning. Supervised learning is further categorised into weakly supervised and semi-supervised methods, whereas unsupervised learning can also take the form of self-supervised learning.

Supervised learning refers to learning from well-curated labelled data often used to learn differences and similarities to automatically group data samples into different categories, e.g., diagnosing normal and abnormal samples of subjects. This is also referred to as classification, in which the model learns to label the data samples according to their corresponding categories. In weakly supervised learning, the dataset is labelled but only at a high level, e.g., in histopathology a WSI with a patient’s level label but without any region-level or cell-level annotations. In semi-supervised learning, a small subset of well-annotated data together with a large amount of unlabelled data are available for training purposes. In unsupervised learning, data is not labelled at all; instead, different similarities and distance metrics are used to find the potential groupings within the data. This is also referred to as data clustering. Self-supervised learning is a form of unsupervised learning where the data itself provides supervision with the help of an auxiliary learning task on a subset or all of the data. In reinforcement learning, learning has a reward or penalty associated with the expected outcome in a particular situation.

### Machine-learning applications in histopathology

The data for machine-learning applications in histopathology involves nuclei, cells, tissues regions, within the whole-slide images, clinical information related to patients' diagnosis, treatments and outcomes, as well as gene expression data [43, 44]. A wide range of digital pathology applications of deep learning include automatic detection, segmentation, and classification of nuclei [32, 45], cells [33, 34] and tissue regions [35] as objects or regions of interest (ROIs). Image segmentation is the process of partitioning image into multiple image segments (ROI) or image objects (nuclei). Manual feature engineering involves quantifying nuclei phenotypes, cellular composition, morphology and orientations [36]. DL pipelines involve automated feature engineering employed for nuclei detection, cell segmentation and classification, as well as tissue segmentation and classification [44]. ML is often used as a postprocessing step after detection and segmentation to make predictions on the downstream tasks of cancer diagnosis [38–40], subtyping [41, 42], patient stratifications in terms of survival, outcome and response to therapy [46, 47].

## AI for immunotherapy in CRC

MSI-H tumours possess more neoantigens and are therefore more visible to the immune system and more responsive to immune-checkpoint inhibitor therapy [4–6]. MSI turned out to be the most popular biomarker out of ten biomarkers explored in these studies on AI for immunotherapy in CRC. However, six out of ten biomarkers were some forms of immune cell quantification in the tumour microenvironment. Below, we provide an introduction to AI-related concepts, followed by a survey of AI literature for immunotherapy in CRC.

**Fig. 3:**
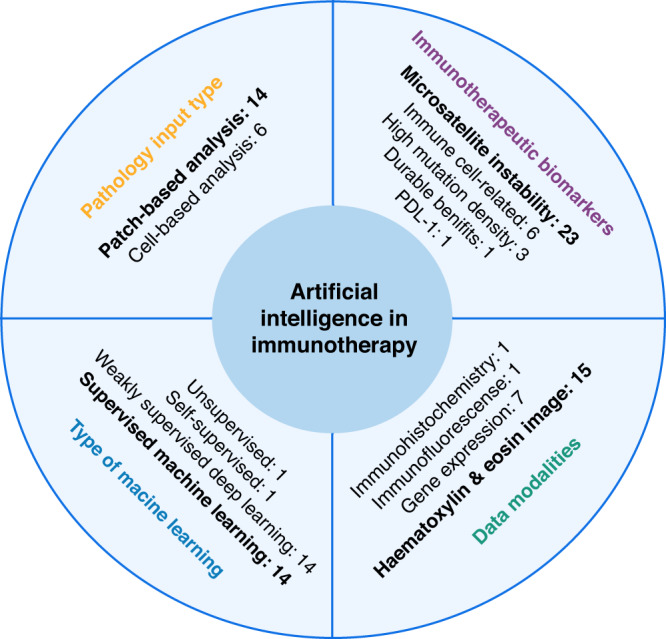
Review summary. Illustration of different key factors considered in a number of AI-based studies categorised in terms of immunotherapeutic biomarkers, data modalities, type of machine learning and pathology input used in the studies.

In Fig. 3, we summarise key factors of our literature review of twenty-six studies of AI predicting immune response-related biomarkers. Twenty-three of these studies predicted MSI status using AI techniques. Since WSIs are large, they are often divided into small image tiles (patches). Patch-based analysis was twice as popular in comparison to cell-based analysis in these studies. Four different data modalities were used involving three different imaging techniques and gene expression data, with H&E images being the most popular data modality. Four different types of machine learning (ML) approaches were employed in these studies with supervised and weakly supervised learning as the most frequently used approach.

Recently, substantial progress has been made in predicting MSI status and immune response-related biomarkers using AI in different data modalities. ML was used for predicting MSI from gene expression data [48–51]. Deep learning (DL) and ML-based approaches were explored for predicting MSI status [52–64], genetic alterations, CRC pathways, and high mutation density (HMD) [55, 59] and tumour mutation burden (TMB) [65] from H&E-stained histology slides. Few of these studies are automatically predicting survival based on immune cell densities directly from H&E image, IHC and gene expression data [66–71]. Details of these studies and their findings are reviewed in the subsequent sections.

### Machine learning for MSI prediction from gene expression data

In this section, we cover studies that employed machine learning for the prediction of MSI status from gene expression data. Chen et al. [48] analysed effects of MSI status on gene expression, and Lu et al. [49] developed an immune-checkpoint inhibition prediction model. Wang et al. [50] and Huang et al. [51] used classical ML techniques to develop “MSIpred” and “MSIseq” for automatic MSI classification, respectively.

SVM appears a widely used classifier for discriminating different classes of MSI status from the gene expression data. Lu et al. [49] developed ML-based models to predict immune-checkpoint-related genetic expressions like TMB, PDL-1, Durable clinical benefit (DCB) or No durable clinical benefit (NDB), and dMMR as digital biomarkers in GI cancers. They used 359 human genes of immunological response to immunotherapy, infiltrating immune cell markers, tumour-specific antigens, tumour markers and essential signalling pathways from RNA profiling data. Among four models, the DCB vs NDB prediction model showed better performance (AUROC = 0.74) than the rest of the immune-checkpoint-related biomarkers, whereas PDL-1 positive vs PDL-1 negative prediction models performed worse (AUROC = 0.52). Other methods [48, 50, 51] have shown better AUROC (0.941–1.0) in predicting MSI-H vs MSI-L and MSI-H vs MSS. MSI status seems better predicted if the model is developed for a single type of GI cancer like CRC and performance is lower when the development cohort consists of data from multiple cancers. The use of different cohorts and different methodologies makes it difficult to compare performance across different studies. The selection of genes for given biomarkers can also have an impact on the model performance.

### Machine learning for predicting immunotherapy-related biomarkers

In this section, we cover studies that aim to evaluate densities of different immune cells and their interplay with tumour epithelial and stromal tissue, their association with overall survival and relevant molecular subtypes. These studies provide similar evidence in favour of different immune cell densities and their significant role in predicting survival independent of the relevant molecular subtypes like MSI.

Väyrynen et al. [66] analysed tumour-associated plasma cells, neutrophils, and eosinophils in tumour intraepithelial and stromal areas within H&E images using classical ML. They found high densities of lymphocytes and eosinophils in tumour–stroma are associated with better survival. They also found higher densities of both intraepithelial & stromal lymphocytes in MSI-H cases.

Gao et al. [67] proposed a novel deep learning framework for the cancer molecular subtype classification. They transformed high-throughput gene expression data of TCGA-CRC cohort into functional spectra using gene set enrichment analysis and achieved balanced accuracy of 90% for consensus molecular subtype (CMS) classification, on 13 validation cohorts (*n* = 3578).

Fujiyoshi et al. [68] hypothesised that T-cell densities (reflecting adaptive anti-tumour immunity) might be inversely associated with tumour budding and poorly differentiated clusters (PDC) in colorectal carcinoma. Their findings suggest that anti-tumour immunity based on cytotoxic T cells may suppress microinvasion. High densities of intraepithelial CD3 + CD8 + and CD3 + CD8 + CD45RO + and cytotoxic T cells are associated with low tumour budding grade, reflecting the suppression of tumour progression by cytotoxic anti-tumour immunity. PDC grade was significantly associated with BRAF mutation, while tumour budding had a tendency towards an inverse association with MSI-high phenotype.

Zhang et al. [69] performed an in-silico analysis of tumour immunity among different MSI statuses in five cancer types, including CRC. They used TIMER to calculate the abundances of immune infiltrates of B cells, CD4 T cells, CD8 T cells, neutrophils, macrophages, and dendritic cells from the gene expression matrix of each TCGA cancer sample. All immune-related gene sets were found to be positively correlated to samples harbouring MSI-H status in CRC. Gene ontology enrichment analysis showed many immune-related function modules to be significantly enriched exclusively in MSI-H CRCs. The distinct infiltration immune cell abundance and immunological gene signatures were stronger predictors of patient survival than MSI status.

Nestarenkaite et al. [70] applied a spatial analysis method which computes immuno-gradient indicators to estimate the migration of immune cells towards the tumour across the tumour/stroma interface. They used HALO multiplex IHC algorithm to detect and extract coordinates of CD8+, CD20+, CD68+ cells. They compute mean cell densities of CD8+, CD20+, and CD68+ in intra-tumoral tissue and within the tumour–stroma interface zone (IZ), which includes tumour, tumour edge and stroma. Independent from molecular characteristics and TNM staging, CD8+ and CD20+ immuno-gradient indicators, which reflect cell migration towards the tumour, were associated with improved patient survival, while the infiltrative tumour growth pattern was linked to worse patient outcome. They found MSI-H shows higher CD8+ and CD68+ cell densities and no difference in of CD20+ in IZ among MSI or MSS.

Karpinski et al. [71] characterised the immune landscape of CRC consensus clusters. They performed unsupervised clustering (*n* = 1492) into five clusters. CRC with tumour purity <0.65 were excluded, rest of the four clusters were mapped to CMS. They reported significant enrichment of innate immune cells (macrophages M0 & M1, activated mast cells and neutrophils) in four of the five clusters. Cluster3–CMS1 displayed both, enrichment of leucocytes related to adaptive immunity and to innate immunity. Cluster2–CMS2 and Cluster5-CMS3 displayed significantly higher epithelial cell content than Cluster3–CMS1 and Cluster4–CMS4.

### Deep learning for MSI prediction directly from H&E images

In this section, we discuss DL approaches for predicting MSI status directly from H&E images. We include only those studies which used H&E-stained routine formalin-fixed and paraffin-embedded (FFPE) diagnostic slides for the prediction of MSI status in CRC cancer.

Kather et al. [52] published a pioneering study to show that DL can predict MSI status directly from histology images in gastrointestinal cancers, including CRC. Several other studies have been published since then. In follow-up multisite validation studies, Echle et al. [53, 64] demonstrated high performance (external validation, *n* = 771 and *n* = 805, AUROC = 0.96) of a DL-based MSI prediction from H&E images. An in-depth analysis of these studies highlights a few key factors that may affect the performance. These factors include having multiple large cohorts covering a wide range of variability and heterogeneity [53], balancing the dataset for MSI and MSS cases at the patient level [52], selecting representative tissue types [54], and weakly supervised learning to select representative tiles [55], using transfer learning or fine-tuning [61] and fine-tuning with self-supervised learning on TCGA-CRC [63], all contributed to performance gains [56–60, 62]. The importance of publicly available data resources like TCGA appears to be another key factor, which not only accelerates the research and development of AI techniques but also promotes reproducibility and head-to-head comparative analysis among different approaches.

**Fig. 4:**
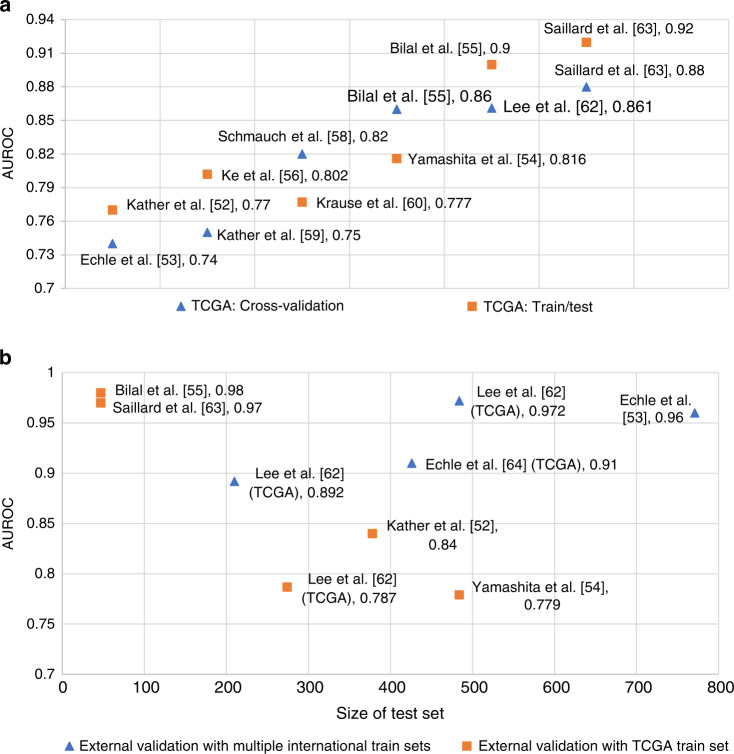
Comparative analysis of published results. Analysing accuracy of different techniques to predict the MSI status from H&E images with the experimental setting of: **a**  cross-validation and  train/test sets of TCGA cohort with AUROC at *y* axis; **b** External validations:  TCGA cohort as a train set and performance on external test sets with AUROC at *y* axis and size of the test sets at *x* axis and  multiple international cohorts combined as a train set and performance on test sets with AUROC at *y* axis and size of the test sets at *x* axis, respectively.

High mutation density (HMD) or tumour mutation burden (TMB) is another immune response-related biomarker; three studies [55, 59, 65] predict HMD directly from the H&E images. Figure 4a shows cross-validation and train/test split performance on the TCGA-CRC cohort. Figure 4b shows external validation performances of different methods.

Despite some outstanding results for prediction of MSI and/or HMD directly from routine diagnostic H&E images, these techniques are still far from adoption in the clinical workflow, which requires the prediction performance to be comparable to the current clinical practice as well as histopathological interpretation and verification of the predictions. To add a degree of interpretability, some recent studies [52–55, 57, 59, 64] have extended their downstream analysis to strongly predicted tiles. Doing it systematically [54, 55, 57] is another important aspect of verifying the existing knowledge and paving a way to future expert-level annotation for such biomarkers in the TME. Yamashita et al. [54] found mucinous annotation as a differential tissue type in addition to tumour epithelium. Bilal et al. [55] found a differential cellular composition of CRC pathways and hypermutated tumours and Cao et al. [57] observed a high proportion of poor differentiation in MSI tumours. Authors in [55] found that tumour-infiltrating lymphocytes (TILs) played a differential role in MSI and HMD tumours.

In summary, large-scale studies with bigger cohorts from multiple clinics, independent external validation, novel learning methodologies, and identification of explainable features are designated key factors for transforming any AI-based biomarker into a potential clinical application.

## Discussion and future of AI in immunotherapy

Among the current trends in the literature, microsatellite instability or MSI has emerged as the most promising AI-based biomarker out of ten different immunotherapeutic biomarkers. Six of these ten biomarkers are related to immune cell quantification in the tumour microenvironment. Immune cells in the tumour microenvironment have been consistently observed as the most relevant histopathological features of MSI tumours in most studies. This suggests a significant role of immune cell quantification in immunotherapy for colorectal cancer.

DL-based techniques appear to have dominated the role of AI in immunotherapy for colorectal cancer. Substantial progress has been made in predicting MSI status and immune response-related biomarkers using AI in different data modalities. SVM appeared as a widely used classical machine-learning method for predicting MSI status from the gene expression data. In gene expression-based analysis, cohort size and selection of right genes for the given biomarkers have shown their impact on the model performance.

The ability to determine MSI status from H&E images is a promising development that is likely to continue. Multiple large developments and validation cohorts covering a wide range of variability and disease heterogeneity, selecting representative tissue types, and using some form of transfer learning, have been evident as the major factors that have improved prediction accuracies besides robustification and generalisation of AI techniques.

For adoption in clinical practice, there must be strong evidence for AI’s performance being at least matching the existing clinical practice on large-scale multi-centric cohorts. Besides, predictions made by AI must have histopathological interpretation and be verifiable. There needs to be a global consensus on the clinical ‘gold standard’ MSI testing and its improved sensitivity and specificity over the currently known values of sensitivity and specificity, which are below 100% [72]. Recent developments in making the prediction models available as open-source software after rigorous testing are encouraging [73, 74]. The open-source TIAToolBox library [73] has made models for MSI [55] and HER2 [75] predictions available for testing by the community. HEAL [74] is another end-to-end tool for histopathology analysis that contains the code for the prediction of MSI and EGFR status.

Several studies have been conducted to predict digital biomarkers related to immunotherapy in an indirect way, e.g., predicting MSI/TMB status, digital profiling of immune cells directly from H&E images and digital quantification or scoring of TILs could be potential immunotherapy biomarkers. With the growth of AI in general and computational pathology in particular, we expect to see further progress in predicting response to immunotherapy directly from H&E images. This would require clinical cohorts of patients with H&E images and data with endpoints like response and outcome to different types of treatments, including immunotherapy. This is an area that has not been explored much through the AI approaches, although its promise could be more widespread than the current framework and approaches of AI in immunotherapy studies. It is also unknown whether AI and histopathology can be used in therapeutic decision-making for alternating immunotherapy approaches, cytokines, oncolytic viruses, tumour vaccines, and other cells in the TME that are not distinguishable from routine histology slides.

Recent developments have demonstrated the utilisation of existing knowledge of the CRC immune landscape, the discovery of potentially novel biomarkers and strategies for immunotherapy. Alderdice et al. [76] have found IL2RB (CD122) as the most common gene associated with immune-checkpoint genes in CRC. Bocciarelli et al. diagnosed rare targetable oncogenic fusion in MSI-High, RAS-BRAF wild-type CRC with MLH1 loss [77]. Another promising direction is the exploration of gene expression and whole exome sequencing data of immune checkpoints, consensus on references and minimal application requirements, as suggested by Barth and Gyorffy [78], combining features from both histology and molecular profiling [79], and particularly novel therapeutic strategies catering CRC heterogeneity [80, 81]. From the “AI in healthcare” perspective, there is good momentum for continuation and further acceleration of research and development to explore these potentially novel directions on the role of AI for immunotherapy in CRC. We anticipate that AI will play a vital role in accelerating the research to explore new biomarkers and the clinical adoption of new methodologies for the stratification of patients likely to benefit from immunotherapy.
